# Role of evolutionary and ecological factors in the reproductive success and the spatial genetic structure of the temperate gorgonian *Paramuricea clavata*

**DOI:** 10.1002/ece3.588

**Published:** 2013-05-08

**Authors:** Kenza Mokhtar-Jamaï, Rafel Coma, Jinliang Wang, Frederic Zuberer, Jean-Pierre Féral, Didier Aurelle

**Affiliations:** 1 Institut National de Recherche Halieutique (INRH)2 rue Tiznit, Casablanca, Morocco; 2 CNRS, UMR 7263 IMBE, Aix-Marseille UniversitéStation Marine d'Endoume, Chemin de la Batterie des Lions, 13007, Marseille, France; 3 Centre d'Estudis Avançats de Blanes (CEAB-CSIC)Accés Cala Sant Francesc 14, 17300, Blanes, Girona, Catalonia, Spain; 4 Institute of Zoology, Zoological Society of LondonRegent's Park, London, NW1 4RY, U.K; 5 CNRS, UMS 3470 Pythéas, Aix-Marseille UniversitéStation Marine d'Endoume, Chemin de la Batterie des Lions, 13007, Marseille, France

**Keywords:** Gamete and larval dispersal, mating system, microsatellites, natural spawning event, parentage analyses

## Abstract

Dispersal and mating features strongly influence the evolutionary dynamics and the spatial genetic structure (SGS) of marine populations. For the first time in a marine invertebrate, we examined individual reproductive success, by conducting larval paternity assignments after a natural spawning event, combined with a small-scale SGS analysis within a population of the gorgonian *Paramuricea clavata*. Thirty four percent of the larvae were sired by male colonies surrounding the brooding female colonies, revealing that the bulk of the mating was accomplished by males from outside the studied area. Male success increased with male height and decreased with increasing male to female distance. The parentage analyses, with a strong level of self-recruitment (25%), unveiled the occurrence of a complex family structure at a small spatial scale, consistent with the limited larval dispersal of this species. However, no evidence of small scale SGS was revealed despite this family structure. Furthermore, temporal genetic structure was not observed, which appears to be related to the rather large effective population size. The low level of inbreeding found suggests a pattern of random mating in this species, which disagrees with expectations that limited larval dispersal should lead to biparental inbreeding. Surface brooding and investment in sexual reproduction in *P. clavata* contribute to multiple paternity (on average 6.4 fathers were assigned per brood), which enhance genetic diversity of the brood. Several factors may have contributed to the lack of biparental inbreeding in our study such as (i) the lack of sperm limitation at a small scale, (ii) multiple paternity, and (iii) the large effective population size. Thus, our results indicate that limited larval dispersal and complex family structure do not necessarily lead to biparental inbreeding and SGS. In the framework of conservation purposes, our results suggested that colony size, proximity among colonies and the population size should be taken into consideration for restoration projects.

## Introduction

Dispersal plays a key role in the spatial partitioning of genetic diversity within and among marine populations and strongly influences the evolutionary dynamics of the populations through natural genetic processes (migration, natural selection, and genetic drift). Mating features, such as individual reproductive success, are also key determinants which contribute to the spatial genetic structure (SGS) of the species.

Reproductive success relies on the rates of fertilization, larval survival, and recruitment, which vary depending on the reproductive strategies, environmental factors, and stochastic events. For marine invertebrate species, releasing one or both set of gametes, fertilization success depends on many factors such as population density, proximity between gamete sources, gamete dispersal, egg size, sperm concentration and longevity, and current regime (see Serrão and Havenhand 2009 for a review). Those factors determine the mating patterns and influence the extent of gene flow through gamete dispersal (Broquet and Petit 2009). The mode of larval development, larval phase duration, larval behavior, larval feeding mode, and larval dispersal also influence gene dispersal. Mating pattern, individual reproductive success, and gamete and larval dispersal may modify the SGS (Epperson and Li 1996), which in return may influence key evolutionary determinants (such as the effective population size) and the mating pattern, for example, by fostering biparental inbreeding (Vekemans and Hardy 2004). Nevertheless, the link between reproductive mode and population structure is not always straightforward (Miller and Ayre 2008). To date, only few genetic studies have emphasized the occurrence of SGS in sessile marine invertebrates at small distances (few centimeters to meters) which were hypothesized to be due to restricted gamete and larval dispersal (Calderón et al. 2007; Blanquer et al. 2009; Ledoux et al. 2010). However, these studies did not examine at the same time the mating patterns, gamete dispersal, and individual reproductive success which may provide a crucial knowledge towards a better understanding of the genesis of such SGS.

The reproductive mode can also have important consequences on population evolution through stochastic effects. Many marine species display high fecundity and high larval mortality which could result in sweepstakes reproductive success: this indicates that only a small subset of the adult population contributes the majority of offspring in the subsequent generation (Hedgecock et al. 2007a). Sweepstakes reproductive success is due to the variance in individual reproductive success through sampling effects either at the fertilization stage or through stochastic larval mortality during the pelagic stage (Hedgecock 1994; Hedgecock et al. 2007b). The sweepstakes reproductive success could generate a “chaotic genetic patchiness” in which the larval pools are heterogeneous (Planes et al. 2002; Hedgecock et al. 2007a) and genetically differentiated from the adult population near where they are sampled (Hellberg 2009). Gonochoric brooding sessile species such as gorgonians are well-suited models to study the genetic impact of reproductive mode as larvae may be easily collected on female colonies after fertilization, allowing an early analysis of reproductive success, although it should be potentially lower for brooding species. Nevertheless, most of the studies on these species dealing with reproductive success have focused on the rate of fertilization during spawning events and the factors that control this rate (e.g., Coma and Lasker 1997a,b; Lasker 2006). These studies have seldom assessed individual reproductive success in spawning events, the factors driving this success and the consequences on the genetic composition of the recruits (but see Coffroth and Lasker 1998; Lasker et al. 2008). The use of highly polymorphic genetic markers such as microsatellites (Avise 2004) together with the development of genetic parentage statistical analysis (Jones et al. 2010) sharpen our insights into mating systems of the species and patterns of dispersal (Selkoe et al. 2006; Christie et al. 2010).

**Figure 1:**
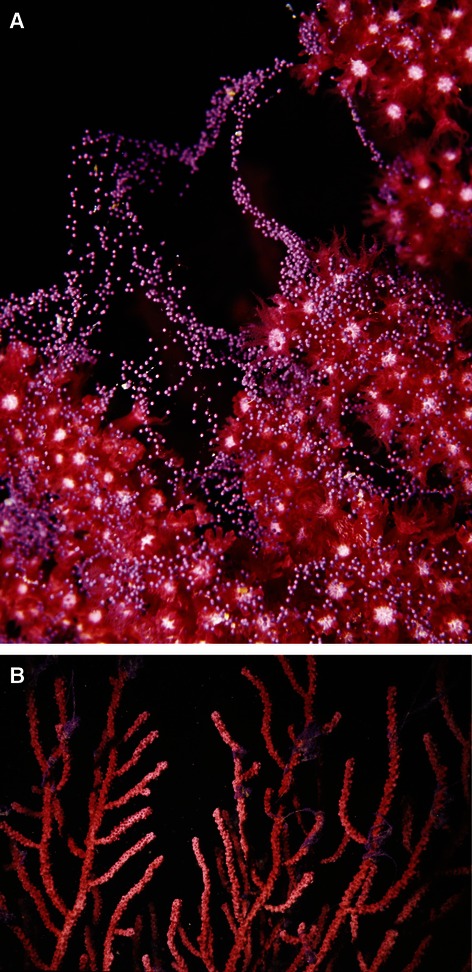
Surface brooding in *Paramuricea clavata*. Just after spawning oocytes and mucus are spread over the surface of female colonies [(A), picture from J.-G. Harmelin]. Short after spawning (min) the oocytes and the mucus form masses mainly on the upper part of the colonies [(B), picture from R. Coma].

The red gorgonian *Paramuricea clavata* (Anthozoa, Octocorallia) is a key species of the highly diverse Mediterranean coralligenous assemblages (True 1970; Ballesteros 2006). This sessile, slow-growing, and long-lived species (Coma et al. 1998; Linares et al. 2007) is widely distributed in the western part of the Mediterranean Sea between 10 m and beyond 200 m depths, with a patchy distribution. The red gorgonian is affected by the combined effects of diving activities (Coma et al. 2004) and mass mortality events linked to the ongoing climate change (Coma et al. 2009; Garrabou et al. 2009). *P. clavata* is a perennial, iteroparous, and gonochoric species without apparent sexual dimorphism (Coma et al. 1995a). The maintenance of the populations relies on sexual reproduction, as asexual reproduction is negligible in this species (Coma et al. 1995a). Female colonies of *P. clavata* are surface brooders that retain eggs through a mucous coating, where embryogenesis takes place (Fig. 1), whereas male colonies release their gametes in the water (Coma et al. 1995a) [also called spermcast mating (Bishop and Pemberton 2006)]. The synchronous spawning occurs in June and July in two or three distinct events lasting each 1–8 days (Coma et al. 1995a). The species generally exhibits parity in sex ratio (Coma et al. 1995a; but see Gori et al. 2007). Sexual maturity is reached when the colony attains 20 cm in height (i.e., an age of around 13 years) and the reproductive effort increases with colony size (Coma et al. 1995b). Once embryogenesis is achieved, the lecitotrophic planula larvae leave the surface of the maternal colony. In aquarium, planulae exhibit a negative phototaxis behavior and the larval phase can span 6 to 23 days. This duration could be overestimated, as planulae did not settle to the substratum in the experiment (Linares et al. 2008a). *In situ*, larvae seem to display a short swimming period after which they settle on the substratum, near the putative maternal colony (Coma et al. 1995a; Linares et al. 2008a). The individual investment in sexual reproduction and fertilization rate are high (Coma et al. 1995b; Linares et al. 2008a). The low and variable recruitment rates (Coma et al. 2001) suggest that the red gorgonian may display a bet-hedging strategy (Slatkin 1974; Linares et al. 2007, 2008a). A previous genetic study on *P. clavata* has evidenced strong genetic differentiation among populations (Mokhtar-Jamaï et al. 2011). Most of the populations exhibited departure from Hardy–Weinberg equilibrium owing to heterozygote deficiencies (Mokhtar-Jamaï et al. 2011). Several factors were hypothesized to explain these heterozygote deficiencies such as null alleles, inbreeding, and Wahlund effect (Mokhtar-Jamaï et al. 2011) but this study did not allow disentangling the contribution of these factors to the observed heterozygote deficiencies.

For the first time, we evaluate individual reproductive success of a marine invertebrate during a natural spawning event along with the SGS of a population. This study was based on paternity assignment of larvae collected on female colonies of *P. clavata* after spawning events. This would help to analyse the variance in reproductive success in this species and the factors affecting this variance, either deterministic (e.g., through differences in height leading to different gonadal output or through distances between colonies) or stochastic (e.g., linked with variable hydrodynamic processes or the inherent stochasticity of fertilization process). The genetic analysis of larvae would also allow estimating the genetic heterogeneity of larval pool, just after fertilization, and its potential impact on genetic structure. Finally, the analysis of SGS among colonies at a local scale may reveal the dispersal abilities of this species and indicate whether sexual reproduction within populations is random or if some inbreeding occurs. The sessile and gonochoric characters of *P. clavata* are well suited for such study as the sexes can be determined on spatially located individuals. Through surface brooding, maternity can be established and eggs can be easily collected in the field.

## Materials and methods

### Study site and field collections

**Figure 2:**
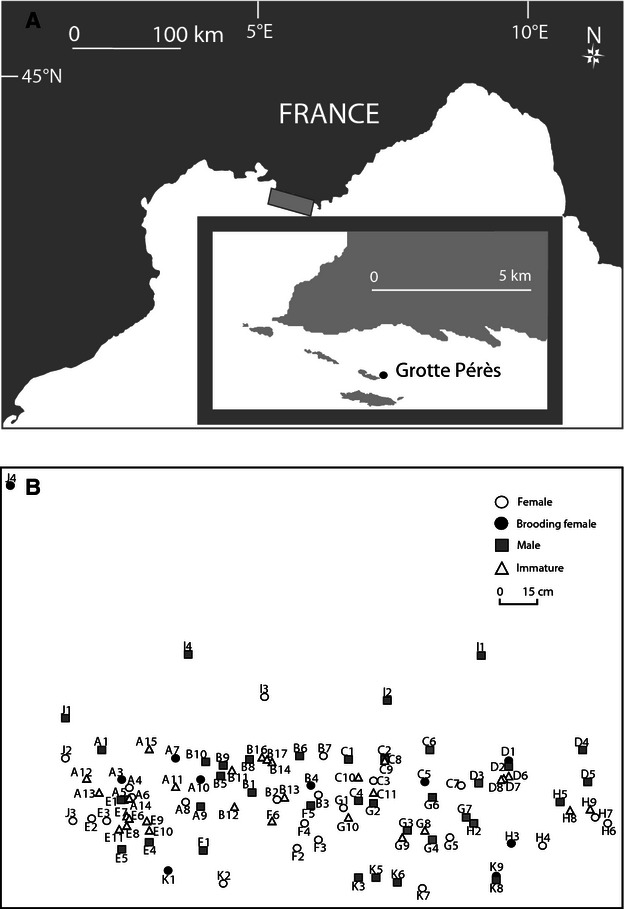
(A) Map of the studied site and (B) localization of the 100 colonies of *Paramuricea clavata*.

The study site was located on Plane Island at 20 m depth (Marseille, France, 43°11′11.40″N, 5°23′28.80″E) (Fig. 2A) where a population of *P. clavata* dwells on a rocky cliff. Within an area of 2 m² (1 × 2 m), the coordinates of all the 87 colonies were determined to generate a map of the study area (Fig. 2B) and the distances between colonies were calculated. The maximum colony heights (distance from the base to the highest apical point) were measured and a 3 cm apical fragment per colony was collected in May 2009 [prior to spawning and corresponding to the period of gonadal ripeness (Coma et al. 1995a)]. The fragment was then divided into two pieces: one piece was fixed in 10% formalin in seawater for sex determination and the other piece was stored in 95% ethanol for genetic analyses. The samples were dissected and the sex was determined by observing gonadal color and appearance under a binocular microscope following Coma et al. (1995a). The sex was only determined for colonies measuring ≥15 cm as sexual maturity in this species is reached around 20 cm height (11–30 cm) (Coma et al. 1995b). Additionally, all colonies larger than 25 cm located in the 3.06 m² neighboring area were also mapped and sampled because of their potential role in fertilization of the central studied area. The sex of these additional colonies was determined and they were added to the study, resulting in a total set of 104 colonies and an overall area of 5.06 m². During June and July 2009, spawning was monitored by SCUBA diving. Oocytes and/or eggs were collected from the upper parts of the surface of 10 female colonies (Table 1), where the oocytes and/or eggs were gathered (Fig. 1), by using 60 mL syringes. They were then transferred to 150 mL glass jars containing 0.2-μm-filtered seawater which was daily replaced. The eggs were allowed to develop into planula larvae during 72 h at ambient seawater temperature (16°C) (Linares et al. 2008a). We randomly selected around 32 larvae per brooding female (Table 1), except for the females A3 and J4 (61 and 74 larvae, respectively) for which two broods were collected during two distinct spawning periods for A3 (A3-1 brood and A3-2 brood, respectively) and two broods were collected from the same spawning period for J4 but with 1 day of interval between collection (J4-1 brood and J4-2 brood, respectively). The larval pool contained larvae from 12 broods. The larvae from the same mother were gathered in the same brood except for A3 and J4 for which the two separate collections were considered separately. Larval DNA was immediately extracted and stored at −20°C for further genetic analyses.

**Table 1:** Number of analyzed larvae per brood and paternity assignments results with COLONY software for the 12 broods

	A3-1	A3-2	A7	A10	B4	C5	D1	H3	J4-1	J4-2	K1	K9	Total	Mean per brood ± SD
Number of analyzed larvae	29	32	30	30	31	30	31	34	36	38	33	31	385	32.08 ± 2.71
Number of assigned larvae	18	12	15	10	20	13	7	5	9	7	6	10	132	11.00 ± 4.77
% of assigned larvae	62.07	37.50	50.00	33.33	64.52	43.33	22.58	14.71	25.00	18.42	18.18	32.26	/	35.16 ± 16.89
Number of assigned father	9	7	9	6	6	6	6	5	6	5	5	7	/	6.42 ± 1.38
% of full-sibling among larvae	7.14	2.62	4.14	2.99	15.91	5.52	1.94	8.73	10.32	12.94	2.65	3.66	**/**	6.55 ± 4.55

### Microsatellite genotyping and identification of multilocus genotypes

Total genomic DNA was extracted from 104 colonies and 385 larvae (Table 1) following a salting-out protocol (Mokhtar-Jamaï et al. 2011). Samples were genotyped at five microsatellite loci: Parcla 09, 12, 14, 17 as described in Molecular Ecology Resources Primer Development Consortium et al. (2010) and Par_d from Agell et al. (2009) but as described in Mokhtar-Jamaï et al. (2011). The 104 colonies were also genotyped at a sixth locus: Parcla 10. In case of mismatches at one locus, between larvae and their mothers (observed at low frequency: 1.5%), the larval genotypes were coded as missing data at this locus.

Given the proximity between some colonies in the field (from 1 cm), which may cause multiple sampling of the same colony, we computed the unbiased probability of identity (*P*_ID_) (Kendall and Stewart 1977) that two sampled individuals share identical multilocus genotypes (MLG) by chance, through sexual reproduction, using GIMLET (Valière 2002). The multilocus *P*_ID_ value was 2.77 × 10^−8^. Four pairs of colonies shared the same MLG and colonies were separated by less than 5 cm in the field. Given the low value of *P*_ID_, each distinct genotype was included only once in the final dataset which contained 100 colonies.

### Size-defined stage classes, reproductive status, and colony sex

We defined stage classes among the colonies based on the maximum colony height and their reproductive status. We classified the sampled colonies into 20 cm size intervals (corresponding to the mean height of sexual maturity in this species), spanning the entire measured size range (from 3 to 92 cm), in order to test for kinship relationships among and within colony stage classes and for temporal structure analysis among colony stage classes. As we were able to determine the sex from colonies of 15 cm, the 29 colonies measuring between 3 and 14 cm were assigned to the first stage class C0 and were assumed as sexually immature. The second stage class C1 was composed of 37 colonies measuring between 15 and 30 cm in height; the third stage class C2 grouped 26 colonies measuring between 31 and 50 cm. Finally, the last stage class C3 gathered all the colonies measuring more than 51 cm, (i.e., 8 colonies). Among the 71 sexually mature colonies (C1, C2, C3), 33 colonies were identified as female and 38 as male. The sample displayed a sex ratio not significantly different from 1:1 (chi-square test: χ² = 0.352; d.f. = 1; *P* = 0.552). For all the following analyses involving the broods only the mature colonies (from C1 to C3) were considered as the putative parental population, whereas all colonies (from C0 to C3) were considered for the analyses among colonies. The details for each colony [sex, size, stage class for kinship relationship, estimation of reproductive output, and male reproductive success (see results)] are given in Table S1.

### Genetic diversity, Hardy–Weinberg equilibrium, and linkage disequilibrium

Observed and Nei's (1973) unbiased expected heterozygosities were computed using GENETIX v.4.05 (Belkhir et al. 2004) and allelic richness [*A*r(g)] and private allelic richness [*A*p(g)] were computed with a rarefaction procedure using HP-RARE (Kalinowski 2005) with the minimum number of genes set to 48 for putative parental population and broods. Null allele frequencies were estimated with FREENA (Chapuis and Estoup 2007). Linkage disequilibrium (LD) between each pair of loci was tested using the permutation procedure (*n* = 1000) implemented in GENETIX. GENEPOP v.4.0 (Rousset 2008) was used to test for Hardy–Weinberg equilibrium for each locus using the score test for heterozygote deficiency and the significance was addressed by a Markov Chain algorithm with default parameters. Single and multilocus Weir and Cockerham's (1984) *f* estimator of *F*_IS_ were computed. Besides to determine whether colonies were more related than expected under panmixia the *R*_XY_ pairwise relatedness coefficient of Queller and Goodnight (1989) was computed among all the colonies and the observed mean and variance of *R*_XY_ were compared with their expected distribution under the null hypothesis of panmixia using 1000 permutations of alleles as implemented in IDENTIX (Belkhir et al. 2002).

### Parentage analyses

The two-generation-pedigree maximum likelihood methods implemented in COLONY v.2.0 software (Wang 2004; Wang and Santure 2009; Jones and Wang 2010) were used to infer sibship and parentage relationships using individual multilocus genotypes. This software has the advantage of considering and partitioning simultaneously all the individuals which is more powerful than pairwise likelihood methods (Jones et al. 2010). For the 385 larvae from the 12 broods (with their known mothers and maternal sibships), paternity and sibship assignments were conducted with the 38 male colonies from the putative parental population as candidate fathers. In order to check the accuracy of parentage and sibship assignments, analyses were also conducted first by setting the maternal relationships (i.e., maternity and maternal sibships) as unknown and then by setting maternal sibships as known but maternity as unknown. Maternity was highly accurately assigned in the case of unknown maternal relationships and completely assigned in the case of known maternal sibships. Paternity assignments were highly concordant between all of the three analyses (i.e., known maternity, known maternal sibship but unknown maternity, and unknown maternal sibship and unknown maternity), verifying the reliability of our parentage and sibship assignments. Kinship relations (half-sibling, full-sibling, and parent–offspring) were also investigated among the 100 colonies divided into four stage classes using males and females belonging to the upper stage class(es) as candidate parents: for C0, parents were considered in C1, C2, and C3 classes; for C1 in C2 and C3 classes; for C2 only the C3 class was considered. COLONY was launched with the null allele frequencies computed with FREENA for the allelic dropout rate and a rate of 0.01 for all other kinds of errors. Five medium length runs were carried out to check for concordance between runs using the full-likelihood method and a medium likelihood precision. We only considered the reliably inferred assignments with a probability of 0.9 or greater for larval paternity assignments. For parentage assignments among colonies, we considered the results from the best maximum likelihood configuration because we were interested in inferring all the ‘true’ relationships among colonies. The effective size of the population was inferred from COLONY outputs under the hypothesis of random mating (Wang 2009).

After paternity assignments, a multiple regression analysis was conducted using STATISTICA software v.6.1 (StatSoft Inc., Tulsa, OK) to establish the relationship between male reproductive success, computed as the number of larvae a male sired, and the main factors that can affect it such as colony height (hereafter height) and the male to female distance (hereafter distance). This relationship was investigated on the basis of each female colony in relation to all male colonies.

### Spatial and temporal genetic structure

#### Bayesian clustering method

We investigated temporal (i.e., among colony stage classes) and spatial population structure among colonies using the Bayesian approach implemented in STRUCTURE v.2.3 (Pritchard et al. 2000; Falush et al. 2003, 2007). Twenty independent runs of 15.10^6^ iterations (burn-in of 150,000) were computed for each *K* value (*K* varying from 1 to 15) under the admixture model, the assumption of correlated allele frequencies among clusters and using the recessive allele option to cope with null alleles (Falush et al. 2007). We plotted the log probability of the data [Ln*P*(D)] as a function of *K* across the 20 runs to select the most likely *K* value (Pritchard et al. 2007). CLUMPP v.1.1 (Jakobsson and Rosenberg 2007) was used to merge the results across the 20 runs for the selected *K* value.

#### Genetic differentiation

We computed Weir and Cockerham's (1984) θ estimator of *F*_ST_. As null alleles can induce an overestimation of genetic distance (Chapuis and Estoup 2007), pairwise *F*_ST_ estimates were also computed following the Excluding Null Allele (ENA) method in FREENA (Chapuis and Estoup 2007). We addressed significance of pairwise genotypic differentiation among the 12 broods and parental population and among the four stage classes with an exact test as implemented in GENEPOP using the default parameters.

#### Spatial autocorrelation

Spatial autocorrelation analyses among colonies were performed with SPAGEDI (Hardy and Vekemans 2002). We used as statistics the Moran's I relationship coefficient (M_I_) (Moran 1948) and the kinship coefficient (φ_ij_) described in Loiselle et al. (1995). Distance categories of the spatial autocorrelation analyses were set given the spatial resolution of the studied area and the number of pairs in each category. We defined distance categories every 5 cm from 0 to 100 cm and every 10 cm from 100 to 351 cm (maximal distance between colonies). The first distance categories were merged because of few pairs in each of them, resulting in two categories: 0–15 cm and 15–25 cm and the last distance categories were also merged for the same reason resulting in two categories: 180–200 cm and 200–351 cm. The 95% confidence intervals of the corresponding coefficients were computed through 10,000 permutations of individual locations.

Significance levels were adjusted using a false discovery rate (FDR) correction for multiple tests (Benjamini and Hochberg 1995) when necessary.

## Results

### Genetic diversity, Hardy–Weinberg equilibrium, and linkage disequilibrium

All loci were polymorphic over the 100 colonies analyzed, with the number of alleles ranging between 7, for Par_d, and 22, for Parcla 10, Parcla 12, and Parcla 14, with a mean value of 16 alleles per locus. Among the broods, the observed and unbiased expected heterozygosities, based on five loci, ranged from 0.50 for J4-1 brood to 0.77 for A3-1 and A3-2 broods and from 0.48 for J4-1 brood to 0.74 for C5 brood, respectively (Table 2). Observed and unbiased expected heterozygosities were equal to 0.69 and 0.70, respectively, for the putative parental population. Among the broods, the allelic richness and private allelic richness varied, respectively, between 6.12 for J4-1 brood and 7.92 for A10 brood and between 0 for C5, H3, J4-1 broods and 0.47 for A10 brood (Table 2). For the putative parental population the allelic richness and private allelic richness were equal to 10.29 and 0.46, respectively. No significant differences in genetic diversity parameters were found between the 12 broods and the putative parental population (Kruskal–Wallis test, *P* = 0.939, *P* = 0.871, *P* = 0.958, and *P* = 0.539 for *H*o, *H*e, *A*r, and *A*p, respectively). For all the colonies, based on six loci, observed and expected heterozygosities were both equal to 0.74. Linkage disequilibrium was found for one pair of loci in sample A10 brood (Parcla 09–Parcla 17) and three pairs of loci in sample B4 brood (Parcla 09–Par_d, Parcla 12–Par_d, and Parcla 12–Parcla 14) after FDR correction; otherwise no global linkage disequilibrium was observed. The multilocus *F*_IS_ value was equal to 0 (not significant at a 5% FDR) for all the colonies, 0.04 (significant at a 5% FDR) for the larval pool, and 0.04 (significant at a 5% FDR) for the larval pool combined to parental population (Table S2, for details per locus). Null allele frequencies varied between 0 for Parcla 09, Parcla 10, Parcla 14, and Par_d and 0.06 for Parcla 12, with a mean value of 0.02 for all the colonies. The mean and variance of *R*_XY_ were respectively equal to −0.01 and 0.04. The mean of *R*_XY_ was not significantly different from the mean expected under panmixia (*P* = 0.577) whereas variance of *R*_XY_ was significantly higher than expected (*P* = 0.042).

**Table 2:** Parameters of genetic diversity for the 12 broods, putative parental population (based on five microsatellite loci) and all the colonies (based on six microsatellite loci)

	Parameter			

Sample	*H*o	*H*e	*A*r	*A*p
Based on 5 loci				
Parental population	0.69	0.70	10.29	0.46
A3-1 brood	0.77	0.68	7.22	0.19
A3-2 brood	0.77	0.67	7.17	0.15
A7 brood	0.71	0.62	6.65	0.06
A10 brood	0.71	0.63	7.92	0.47
B4 brood	0.66	0.6	7.39	0.19
C5 brood	0.71	0.74	6.82	0
D1 brood	0.66	0.60	7.24	0.07
H3 brood	0.73	0.61	6.14	0
J4-1 brood	0.50	0.48	6.12	0
J4-2 brood	0.57	0.51	7.12	0.30
K1 brood	0.68	0.67	7.46	0.27
K9 brood	0.55	0.49	6.36	0.13
Based on 6 loci				
All colonies	0.74	0.74	14.82	/

*H*o, observed heterozygosity; *H*e, unbiased expected heterozygosity; *A*r and *A*p, rarefied allelic and private allelic richness, respectively.

### Larval paternity analyses

Paternity was assigned for 132 larvae out of 385 (34.3%). On an average 35.2 ± 16.9% of larvae per brood were assigned a father among the 38 sampled male colonies (Table 1). Multiple paternity was observed in all the broods (Table 1) with on average 6.4 ± 1.4 (mean ± SD) different assigned fathers per brood (Table 1). Out of the 38 male colonies, 20 males were identified as fathers at least once (52.6%). A male that sired was on average involved in 6.6 ± 4.7 fertilization events. All those males (except G7 and H5 which were assigned as father only once) were involved in fertilization events with several females, on average with 3.9 ± 1.8 different females. Almost all the colonies that mate were not kin related (data not shown). The distance traveled by a successful male gamete computed from these data ranged between 2 cm and 323 cm with a median distance of 98 ± 69.6 cm. The percentage of full sibship within each brood varied between 1.9% for D1 brood and 15.9% for B4 brood with a mean value of 6.6 ± 4.6% (Table 1). For the whole larval pool, the percentage of full sibship was 5% and the percentage of paternal half sibship was 1.8%. Thirty-six additional males were needed to account for the unassigned larvae and the inferred effective population size was 29 colonies (95% CI: 18–47 colonies).

**Figure 3:**
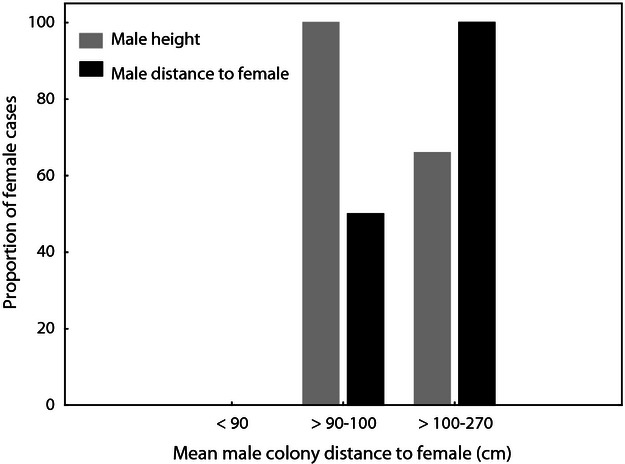
Summary of the results of multiple regressions. Proportion of female colonies on which male height and male distance to female colonies had significant effect as a function of the mean distance classes between male and female colonies (cm).

Male reproductive success, ranging from 0 to 19 (Table S1), was affected by male height and male to female distance (Fig. 3; Table S3). In order to search for a pattern explaining the observed male reproductive success (Table S3), we computed the distance range of each male colony to each female colony (range, minimum distance, maximum distance, and mean distance). The mean male colony distance to female appeared as the most integrative value. Then, we organized the data according to the mean distance parameter. The pattern that comes out was the one shown in Figure 3. The effect of male height was to increase male reproductive success with increasing male colony height whereas the effect of distance was to decrease male reproductive success with increasing distance (Table S3). However, the contribution of these factors to male reproductive success varied as a function of the mean distance of all male colonies to each female which ranged between 63 and 270 cm (Fig. 3). Neither of both factors affected male reproductive success within the close proximity to the female colonies (i.e., <90 cm) for none of the female colonies. At a 90–100 cm distance range, height became the main factor affecting male reproductive success contributing to explain male success on all the female colonies. Beyond 100 cm distance, the contribution of the height factor to male success diminished, exhibiting an effect for only 66% of the female colonies (Fig. 3). The contribution of the distance factor to the male success exhibited a complementary pattern to that of the height factor. The distance factor increased its contribution to the male success from significantly affecting male success on 50% of the female colonies (at the 90–100 cm distance range) to contributing to explain male success on all colonies placed at distance beyond 100 cm (Fig. 3).

### Parentage relationship among colonies

Over the 100 colonies, 98% of colonies (i.e., all except C10 and J1) were involved in at least one parentage relationship (full and half sibship or parent–offspring relationship) and 18% and 98% of colonies were involved in a full sibship and a half sibship, respectively. The details of parentage assignments per stage class are given in Table 3. Fourteen mother–offspring dyads involving six different mothers and eleven father–offspring dyads involving five different fathers were inferred. For each mother and father 2.3 ± 1 and 2.2 ± 0.8 offspring were identified, respectively.

**Table 3:** Results of parentage assignments with COLONY software among the 100 colonies

	CO	C1	C2	C3
% of full sibship	0.49	0.90	0.31	0
% of colonies involved in a full sibship	13.79	32.43	7.69	0
% of maternal half sibship	5.91	5.56	7.69	14.29
% of colonies involved in a maternal half sibship	86.21	89.19	96.15	62.50
% of paternal half sibship	6.40	5.26	6.77	14.29
% of colonies involved in a paternal half sibship	96.55	94.59	92.31	62.50
% of half sibship	12.31	10.82	14.46	28.58
% of colonies involved in a half sibship	96.55	100	100	87.50
Number of mother–offspring dyads			14	
Number of father–offspring dyads			11	

### Spatial and temporal structure

The Bayesian clustering method did not reveal neither spatial nor temporal genetic discontinuity among all the colonies as only one cluster was detected with STRUCTURE (Fig. S1).

Over all loci *F*_ST_ value was 0.14 when comparing the 12 broods, while it was 0.12 when comparing the 12 broods and putative parental population. The corresponding differentiation test was highly significant. Pairwise *F*_ST_ values between pairs of broods ranged from 0.006 for A3-1 versus A3-2 and J4-1 versus J4-2 to 0.212 for C5 versus K9 whereas pairwise values between pairs of broods and the putative parental population ranged from 0.037 with A3-2 to 0.107 with C5 (Table 4). No significant differences were observed between pairwise *F*_ST_ and pairwise *F*_ST_ corrected for null alleles (*t*-test, *P* = 0.884). All pair-wise *F*_ST_ between broods were significant (after FDR correction) except A3-1 versus A3-2 and J4-1 versus J4-2 which corresponded to two different broods collected from the same female colony (A3 and J4, respectively). Pairwise *F*_ST_ values between the four stage classes varied between −0.011 (C1 vs. C3) and 0.001 (C0 vs. C1 and C0 vs. C3) and were not significantly different from pairwise *F*_ST_ corrected for null alleles (*t*-test, *P* = 0.618). All pairwise values between stage classes were not significant after FDR correction, suggesting a lack of temporal structure among colonies within the studied site.

**Table 4:** Pairwise *F*_ST_: (A) among parental population and broods (based on five microsatellite loci) and (B) among the four stage classes (based on six microsatellite loci)

(A)	Parental population	A3-1	A3-2	A7	A10	B4	C5	D1	H3	J4-1	J4-2	K1	K9
Parental population	**–**	**0.050**	**0.037**	**0.056**	**0.056**	**0.055**	**0.107**	**0.058**	**0.066**	**0.075**	**0.070**	**0.079**	**0.080**
A3-1		**–**	0.006	**0.095**	**0.077**	**0.096**	**0.155**	**0.097**	**0.121**	**0.161**	**0.144**	**0.089**	**0.196**
A3-2			**–**	**0.082**	**0.073**	**0.066**	**0.170**	**0.116**	**0.120**	**0.159**	**0.144**	**0.099**	**0.176**
A7				**–**	**0.102**	**0.130**	**0.187**	**0.155**	**0.079**	**0.189**	**0.180**	**0.146**	**0.207**
A10					**–**	**0.119**	**0.167**	**0.137**	**0.104**	**0.157**	**0.164**	**0.126**	**0.199**
B4						**–**	**0.193**	**0.161**	**0.155**	**0.195**	**0.184**	**0.130**	**0.201**
C5							**–**	**0.150**	**0.174**	**0.206**	**0.204**	**0.154**	**0.212**
D1								**–**	**0.172**	**0.086**	**0.073**	**0.115**	**0.117**
H3									**–**	**0.180**	**0.178**	**0.150**	**0.210**
J4-1										**–**	0.006	**0.140**	**0.078**
J4-2											**–**	**0.134**	**0.091**
K1												**–**	**0.193**
K9													**–**

(B)	CO	C1	C2	C3
CO	**–**	0.001	−0.007	0.001
C1		**–**	−0.001	−0.011
C2			**–**	−0.005
C3				**–**

Significant values after false discovery rate (FDR) are in bold.

**Figure 4:**
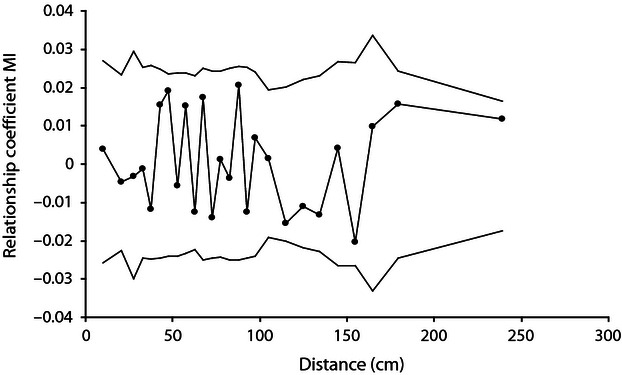
Correlogram (—•—) of the spatial autocorrelation analysis of Moran's I relationship coefficient (M_I_) over 26 distance categories. The 95% confidence intervals are represented.

The spatial autocorrelation analyses failed to detect any spatial structure over all the distance categories as M_I_ and φ_ij_ values were not significant and the correlograms were confined within the 95% confidence intervals of no association between geographical distances and pairwise genetic comparisons between individuals (Fig. 4 for M_I_ correlogram, a similar correlogram was obtained for φ_ij_ data not shown).

## Discussion

### Mating system

The present study showed that about a third of *P. clavata* planulae were sired by males surrounding the brooding female colonies within 5 m^2^. This result suggests that a part of mating takes place among colonies within close proximity but can extend beyond this area as all the putative fathers were not identified. Indeed, considering our exhaustive sampling scheme of the studied area and the high detection power allowed by microsatellites, the nonidentified fathers should be present outside of this area. The presence of alleles in the larval pool that did not match any of the examined male genotypes (data not shown) is a parallel source of evidence supporting this conclusion. At a larger spatial scale (400 m²), similar results were encountered on the surface brooding octocoral *Pseudopterogorgia elisabethae* in the Caribbean (Lasker et al. 2008).

Male height [size as a proxy of colony gonadal output, which increases with colony size (Coma et al. 1995b)] and male distance to the brooding females contributed to explain differences in male reproductive success in *P. clavata*. Male success increased with male height and decreased with increasing male to female distance. This is congruent with several previous studies that reported a decrease in fertilization success and individual reproductive success among broadcast and spermcast spawning species with increasing distance between males and females (Brazeau and Lasker 1992; Yund and McCartney 1994; Levitan and Petersen 1995; Coma and Lasker 1997a,b; Coffroth and Lasker 1998). Sperm dilution at the scale of few meters has also been documented to strongly reduce fertilization in some gorgonian species (Coma and Lasker 1997a,b). However, neither distance effect (with distance ranging from 1 to 20 m) nor male height effect were observed for *P. elisabethae* (Lasker et al. 2008). The differences between both studies could be related to: (a) differences in male density between the two studies (0.2 male/m² for *P. elisabethae* vs. 7.6 males/m² for *P. clavata*), (b) the clear pattern of increase on gonadal production with colony height exhibited by *P. clavata*, a pattern that is much less evident in *P. elisabethae*, and (c) the higher production of gonads in *P. clavata* in contrast to that in *P. elisabethae* (0.08 mm^3^/polyp vs. 0.28 mm^3^/polyp in females and 0.20 mm^3^/polyp vs. 0.57 mm^3^/polyp in males, respectively for *P. elisabethae* and *P. clavata*; species with similar gonad size) (Coma et al. 1995b; Gutiérrez-Rodríguez and Lasker 2004). Indeed, sperm competition has been documented to be more intense at high male density whereas, at low male density all males may have a similar probability to mate any female if sperm dispersal is not limited and if the distribution of male–female distances is not skewed (Kokko and Rankin 2006). Likewise, under high densities, large males that produce more gametes have higher probabilities to mate than smaller ones that produce less gametes, as reproductive effort increases with colony size (Coma et al. 1995b). The occurrence of males outside the studied area that accomplished the main bulk of the mating could be interpreted as a paucity of nearby sperm. However, the commonly high density of the species (Linares et al. 2008b) together with its pattern of investment in sexual reproduction (Coma et al. 1995b) are consistent with the reported high fertilization rates for this species (Linares et al. 2008a) and could suggest that *P. clavata* may be among the less affected species by sperm limitation.

Despite the male size and the male to female distance effects reported in this study, we observed multiple paternity in all the analyzed broods. However, our estimate of the number of males that contribute to a brood is most probably a lower bound estimate because of: (a) the low number of larvae analyzed per brood compared to the mean number of eggs produced by a mean size female colony (around 33.4 × 10^4^ eggs; R. Coma, unpubl. data), (b) the high fertilization rates observed in *P. clavata* (Linares et al. 2008a), (c) the genetic differentiation among broods, and (d) the large proportion of unassigned larvae. Multiple mating has been emphasized in numerous broadcast and spermcast spawning marine invertebrates (e.g., Coffroth and Lasker 1998; Bishop et al. 2000; Ayre and Miller 2006; Johnson and Yund 2007; Lasker et al. 2008). Although no direct benefits from multiple mating (such as nuptial gifts, nutrient delivery, territorial defense, paternal care) are expected in these mating systems, because it is a consequence of broadcast and spermcast mating systems, it may carry some indirect genetic benefits (Jennions and Petrie 2000; Neff and Pitcher 2005) such as the (i) avoidance of genetic incompatibility (Tregenza and Wedell 2000; Marshall and Evans 2005), (ii) sperm competition (the “good sperm-good genes” and “sexy sons” hypotheses) (Yasui 1997; Kokko et al. 2006), and (iii) pre or postfertilization female choice (Pemberton et al. 2003). In this framework, multiple mating would carry a benefit if females can control the paternity and if the paternity is skewed towards a favorite male. In the absence of such mechanisms, multiple mating to increase brood genetic diversity is the most likely benefit (Johnson and Yund 2007; McLeod and Marshall 2009). In an experimental study, McLeod and Marshall (2009) demonstrated that multiple mating increased offspring performance by enhancing genetic diversity. The increase in brood genetic diversity by multiple mating may have several adaptive values. Multiple mating enables to avoid inbreeding (Foerster et al. 2003) which may be a problematic issue for sessile species with limited larval dispersal. The increase in genetic diversity among offspring may also decrease sibling competition in case of local recruitment (Forsman et al. 2007; Kamel et al. 2010). Multiple mating may be advantageous if half-sibs display cooperative or compensatory interaction (Yasui 1998). Multiple mating has also been suggested to enhance fertilization success (Evans and Marshall 2005). Finally, in unpredictable environment, a genetically diversified brood may ensure the survival of at least a part of the offspring (Forsman et al. 2008).

Surface brooding has been suggested to represent an adaptation that enhances fertilization success by increasing the time of oocyte exposure to sperm (Lasker 2006; Lasker et al. 2008; Linares et al. 2008a). This mode of reproduction also increases the opportunity for multiple mating and therefore it contributes to enhance genetic diversity of the brood. However, all the previous hypotheses should be evaluated for *P. clavata*.

### Family structure in space and time

Most of the colonies at the studied site were linked by a parentage relationship (18% and 98% of the colonies related by full-sib and half-sib relationships, respectively). Moreover, 25 parent–offspring dyads were detected which implies a strong level of self-recruitment (25%) at small scale. The pattern of multiple mating led to the local recruitment of about two offspring per colony. Therefore, the present study revealed the occurrence of a complex level of family structure. This result is in agreement with: (a) the negative phototaxis behavior of the larva and its short swimming period (Linares et al. 2008a), (b) previous *in situ* observations of larval settlement near the maternal colony soon after larval release (Coma et al. 1995a; Linares et al. 2008a), and (c) the limited effective larval dispersal among populations (Mokhtar-Jamaï et al. 2011). Such a strong family structure at small scale has also been underlined in the octocoral *Corallium rubrum* (Ledoux et al. 2010), which reinforces the current view that self-recruitment may be more common than previously thought, even for species with potential larval dispersal (Jones et al. 2005; Almany et al. 2007). However, it is unknown whether or not the occurrence of such family structure in *P. clavata* population has an adaptive value through half-sib cooperative or compensatory interaction (Yasui 1998) or if it is a by-product of other life history traits of this species affected by different selective factors. The local recruitment might also be a strategy to increase the probability of recruiting to a suitable habitat (Strathmann et al. 2002).

The autocorrelation analyses did not reveal any association between genetic relatedness and spatial distance among colonies over 5 m^2^, suggesting either that neighborhood size was likely to be equal or greater than the studied area or that no SGS within populations occurs in this species. Besides, the Bayesian clustering approach did not detect any structure. Given the family structure found at this site, several factors may hinder the occurrence of genetic structure at this fine scale in *P. clavata* such as (i) larvae may randomly settle within the site where free available space is found within the area and thus “larva” shadows [by analogy to “seed shadow” (Levin et al. 2003)] may overlap, causing the distributions of different half-sib families to overlap, therefore reducing the relatedness among adjacent colonies or (ii) thinning processes may occur after larval settlement owing to larval mortality which weakens allele aggregation (Linares et al. 2008b; see Asuka et al. 2005 for a review). These hypotheses have also been considered to explain the absence of isolation by distance (IBD) in the brooding coral *Seriatopora hystrix* (Maier et al. 2009). Our finding contrasts with the occurrence of IBD at small scales documented on recent studies of several Mediterranean sessile invertebrates with similar life history traits (e.g., *Crambe crambe* Calderón et al. 2007; *Scopalina lophyropoda* Blanquer et al. 2009; *Corallium rubrum* Ledoux et al. 2010), indicating that different ecological and evolutionary processes may be operating at these scales within these species to explain their observed genetic structure. Nevertheless, the lack of IBD has also been observed at larger scales for the two self-compatible hermaphrodite corals *Seriatopora hystrix* (Maier et al. 2009) and *Goniastrea favulus* (Miller and Ayre 2008), suggesting that the reproduction mode is not always a good predictor of population genetic structure.

The absence of temporal genetic structure at the studied site is consistent with the rather large effective population size inferred from larval paternity assignment [29 colonies (95% CI: 18–47 colonies); Ne/N ∼ 0.4] and suggests that sweepstakes effects did not occur in the studied population (Hedgecock et al. 2007a,b; Hedgecock 2010). Indeed, while some colonies did not apparently reproduce during the surveyed spawning event, some of them were identified as fathers and mothers in the parentage analyses with the size-defined stage classes. This implies that some males and females reproduce at different spawning events. The genetic differentiation observed between the broods and the putative parental population may be mainly related to the presence in the parental sample of (i) nonbrooding females, (ii) males that did not reproduce, and (iii) to the contribution of external fathers in the broods. These results are consistent with the previously discussed pattern of multiple paternity. The sharing of the same mother at the brood level may also contribute to this genetic differentiation, as the allele frequencies in the brood may then be quite different from the parental population as a whole.

The inbreeding coefficient and the mean value of pairwise relatedness were not significantly different from that expected under panmixia, suggesting a random mating pattern. However, the variance was significantly higher than expected. This result may be due to the numerous full-sib and half-sib relationships uncovered in our sample because the distribution of variance was skewed towards the high relatedness values, suggesting a moderate level of inbreeding. Several factors may have contributed to explain the lack of biparental inbreeding in our study such as (i) the fact that sperm limitation appears to be of limited relevance at the studied scale, (ii) the multiple paternity, and (iii) the large effective population size. The results on inbreeding disagree with early expectations that limited larval dispersal should lead to biparental inbreeding in brooding species (Carlon 1999). However, they agree with random mating observed in several brooding species with limited larval dispersal such as the sponge *Scopalina lophyropoda* (Blanquer et al. 2009), the gorgonian *P. elisabethae* (Lasker et al. 2008), and the corals *Acropora palifera* (Ayre and Miller 2006) and *Seriatopora hystrix* (Maier et al. 2009), indicating that limited larval dispersal and strong population structure do not necessarily lead to biparental inbreeding.

Given the moderate level of inbreeding and the lack of spatial and temporal genetic structures at the studied site, we hypothesize that the heterozygote deficiencies found within other locations at larger scales (Mokhtar-Jamaï et al. 2011) may be due to a sampling effect among different families which could create a spatial Wahlund effect. This would be coherent with the genetic differentiation observed between the broods as demonstrated here for the first time in this species. This study could be extended in other populations of *P. clavata* to further investigate this hypothesis.

### Implication of our results for the conservation of the red gorgonian

The results of the present study provide useful and complementary information for the conservation of the red gorgonian. The achievement of mating among colonies close to each other, implying that mating partly takes place locally, and the occurrence of self-recruitment suggest that the functioning of *P. clavata* populations is mainly driven by local processes. These results confirm the previous recommendations for conservation plans at local scales (Mokhtar-Jamaï et al. 2011). However, the interaction between local processes and the supply of long-distance immigrants within populations (Mokhtar-Jamaï et al. 2011) remains to be studied.

*Paramuricea clavata* populations are affected by the combined effects of mass mortality events (Coma et al. 2009; Garrabou et al. 2009) and anthropogenic activities (Coma et al. 2004; Linares et al. 2010). The methodological basis for a restoration approach has been undertaken on red gorgonian populations (Linares et al. 2008c). The results of the present study are fundamental for the appropriate design and implementation of such restoration project. The male height and male distance to female colony effects suggest that transplanted colonies should be from large colonies and that the colonies should be transplanted close to each other. A sufficient number of colonies should be transplanted within restoration sites in order to yield a large effective population size and to ensure the opportunity of multiple mating. If the half-sib family structure uncovered in this study is a common trait in *P. clavata* populations and if it may have an adaptive value, it should be taken into consideration for restoration project. However, the hypothetical “role” of half-sib family structure within populations should be first investigated.

## Author Information Box

This article was part of the PhD work of K. Mokhtar-Jamaï, who focused on the application of genetic approaches to study the population structure, mating system and dispersal in *Paramuricea clavata* for conservation purposes. R. Coma is a researcher interested in understanding the structure and functioning of marine ecosystems. J. Wang is a population geneticist interested in developing genetics models and methods of analysis of empirical data to address issues in evolutionary and conservation biology. F. Zuberer is in charge of the diving service of the OSU Pythéas and is interested in marine biodiversity. J.-P. Féral is a researcher interested in ecology and evolution of marine biodiversity. D. Aurelle is an associate professor interested in evolutionary biology, population genetics, and adaptive processes.

## Supporting Information

**Table S1.** Information about the examined colonies (*N* = 100): sex (M = male, F = female, I = Indeterminate); size (maximum height in cm); colonies' stage class for kinship relationship (C0 = <15 cm, C1 = 15–30 cm, C2 = 31–50 cm, C3 = >51 cm); estimated reproductive output [computed on the basis of the biometry and gonadal production of the colonies (Coma et al. 1995a,b, 1998)]; male reproductive success (i.e., the number of larvae that each male sired)

**Table S2.** (a) f estimator of *F*_IS_ for the larval pool, the larval pool combined with parental population and all the colonies (in bold significant values after FDR); (b) null allele frequency per locus for all the colonies

**Table S3.** Multiple regression analysis to examine whether colony height (height) and male to female distance (distance) contributed to explain male reproductive success estimated with the surface brooding larvae of each distinct female colony (results from J4-1 and J4-2 were grouped as larvae belong to the same spawning). (a) Slopes (± SE) for the different variables, intercept (A), adjusted squared r (Adj R^1^). (b) Partial regression coefficients. Not significant (ns), ****P* < 0.001, ***P* < 0.01, **P* < 0.05

**Figure S1.** Plot of LnP(D) as a function of the number of clusters (K) across the 20 runs.
