# Supplementary material for: Role of evolutionary and ecological factors in the reproductive success and the spatial genetic structure of the temperate gorgonian Paramuricea clavata
Source: Ecol Evol. 2013 May 8;3(6):1765–79. doi: 10.1002/ece3.588 (PMC3686208; doi:10.1002/ece3.588)
Supplement: Supplementary file 1 [file ece30003-1765-SD1.pdf]

Role of evolutionary and ecological factors in the reproductive success and the spatial genetic structure of the temperate gorgonian *Paramuricea clavata*

ONLINE SUPPORTING INFORMATION

K. MOKHTAR-JAMAÏ*[+], R. COMA[‡], J. WANG[†], F. ZUBERER[§], J.-P. FÉRAL[+], D. AURELLE[+]

* *Institut National de Recherche Halieutique (INRH), 2 rue Tiznit Casablanca Morocco,* [+] *Aix-Marseille Université, CNRS, UMR 7263 IMBE, Station Marine d'Endoume, Chemin de la Batterie des Lions, 13007 Marseille, France,* [‡] *Centre d'Estudis Avançats de Blanes (CEAB-CSIC), Accés Cala Sant Francesc 14, 17300 Blanes, Girona, Catalonia, Spain,*

[†] *Institute of Zoology, Zoological Society of London, Regent's Park, London NW1 4RY, UK,*

[§] *Aix-Marseille Université, CNRS, UMS 3470 Pythéas, Station Marine d'Endoume, Chemin de la Batterie des Lions, 13007 Marseille, France.*

Correspondence: Kenza Mokhtar-Jamaï / Didier Aurelle ; E-mail: mokhtarjamaikenza@hotmail.com / didier.aurelle@univ-amu.fr

Table S1: Information about the examined colonies (N=100): **sex** (M = male, F= female, I= Indeterminate); **size** (maximum height in cm); **colonies' stage class for kinship relationship** (C0= <15 cm, C1= 15-30 cm, C2= 31-50 cm, C3= >51 cm); **estimated reproductive output** [computed on the basis of the biometry and gonadal production of the colonies (Coma *et al.* 1995 a, b, 1998)]; **male reproductive success** (i.e., the number of larvae that each male sired)

Colony	Sex	Size	Stage class for kinship relationship	Estimated Reproductive output	Reproductive success
A1	M	18	C1	53398	3
A3	F	30	C1	70730	/
A4	F	36	C2	183430	/
A5	M	16	C1	43760	3
A6	F	27	C1	59194	/
A7	F	43	C2	308933	/
A8	F	22	C1	44926	/
A9	M	21	C1	106817	0
A10	F	23	C1	48431	/
A11	I	12	C0	/	/
A12	I	8	C0	/	/
A13	I	6	C0	/	/
A14	I	8	C0	/	/
A15	I	5	C0	/	/
B1	M	40	C2	451962	7
B2	F	17	C1	5719	/
B3	F	41	C2	285041	/
B4	F	72	C3	713129	/
B5	M	20	C1	63805	3
B6	M	22	C1	115553	0
B7	F	20	C1	7527	/
B8	M	19	C1	58507	0
B9	M	35	C2	359369	3
B10	M	17	C1	48481	0
B11	I	6	C0	/	/
B12	I	5	C0	/	/
B13	I	14	C0	/	/
B14	I	6	C0	/	/
B16	I	4	C0	/	/
B17	I	4	C0	/	/
C1	M	26	C1	138517	5
C2	M	16	C1	43760	3
C3	F	19	C1	6902	/
C4	M	20	C1	63805	0
C5	F	40	C2	219179	/
C6	M	30	C1	176415	11
C7	F	29	C1	66792	/
C8	M	15	C1	40361	0
C9	I	8	C0	/	/
C10	I	12	C0	/	/
C11	I	13	C0	/	/
D1	F	34	C2	166545	/
D2	M	18	C1	53398	0
D3	M	46	C2	662358	10
D4	M	40	C2	451962	15

D5	M	15	C1	40361	0
D6	I	11	C0	/	/
D7	I	11	C0	/	/
D8	I	11	C0	/	/
E1	M	31	C2	292729	0
E2	F	33	C2	158351	/
E3	F	36	C2	183430	/
E4	M	35	C2	359369	0
E5	M	17	C1	48481	0
E6	I	3	C0	/	/
E7	I	10	C0	/	/
E8	I	6	C0	/	/
E9	I	5	C0	/	/
E10	I	7	C0	/	/
E11	I	3	C0	/	/
F1	M	23	C1	124569	0
F2	F	18	C1	6299	/
F3	F	24	C1	52043	/
F4	F	18	C1	6299	/
F5	M	25	C1	143419	0
F6	I	13	C0	/	/
G1	F	39	C2	209999	/
G2	M	40	C2	451962	3
G3	M	16	C1	43760	0
G4	M	16	C1	43760	0
G5	F	20	C1	7527	/
G6	M	58	C3	936532	0
G7	M	22	C1	115553	1
G8	I	14	C0	/	/
G9	I	4	C0	/	/
G10	I	12	C0	/	/
H2	M	17	C1	48481	0
H3	F	32	C2	150326	/
H4	F	50	C2	402589	/
H5	M	18	C1	53398	1
H6	F	29	C1	66792	/
H7	F	17	C1	5719	/
H8	I	10	C0	/	/
H9	I	11	C0	/	/
I1	M	61	C3	1019851	10
I2	M	35	C2	359369	7
I3	F	38	C2	200980	/
I4	M	63	C3	1076998	8
J1	M	63	C3	1076998	8
J2	F	56	C3	466352	/
J3	F	33	C2	158351	/
J4	F	92	C3	1079139	/
K1	F	50	C2	402589	/
K2	F	33	C2	158351	/
K3	M	26	C1	138517	19
K5	M	34	C2	342188	0
K6	M	35	C2	359369	5
K7	F	38	C2	200980	/
K8	M	60	C3	991757	7
K9	F	36	C2	183430	/

Table S2. (a) f estimator of F_{IS} for the larval pool, the larval pool combined with parental population and all the colonies (in bold significant values after FDR); (b) null allele frequency per locus for all the colonies

(a) Fis

sample \ locus	Parcla 09	Parcla 10	Parcla 12	Parcla 14	Parcla 17	Par_d	Over all loci
Based on 5 loci:							
Larval pool	0.08	/	**0.13**	-0.02	-0.06	0.05	**0.04**
Larval pool and parental population	0.06	/	**0.13**	-0.02	**-0.05**	0.05	**0.04**
Based on 6 loci							
Whole colonies	-0.04	-0.07	0.14	-0.04	**0**	0	0

(b) Null allele frequencies

sample \ locus	Parcla 09	Parcla 10	Parcla 12	Parcla 14	Parcla 17	Par_d	Over all loci
Based on 6 loci:							
Whole colonies	0	0	0.06	0	0.03	0	0.02

Table S3. (a) f estimator of F_{IS} for the larval pool, the larval pool combined with parental population and all the colonies (in bold significant values after FDR); (b) null allele frequency per locus for the all the colonies

(a) Fis

sample \ locus	Parcla 09	Parcla 10	Parcla 12	Parcla 14	Parcla 17	Par_d	Over all loci
Based on 5 loci:							
Larval pool	0.08	/	**0.13**	-0.02	-0.06	0.05	**0.04**
Larval pool and parental population	0.06	/	**0.13**	-0.02	**-0.05**	0.05	**0.04**
Based on 6 loci							
Whole colonies	-0.04	-0.07	0.14	-0.04	**0**	0	0

(b) Null allele frequencies

sample \ locus	Parcla 09	Parcla 10	Parcla 12	Parcla 14	Parcla 17	Par_d	Over all loci
Based on 6 loci:							
Whole colonies	0	0	0.06	0	0.03	0	0.02

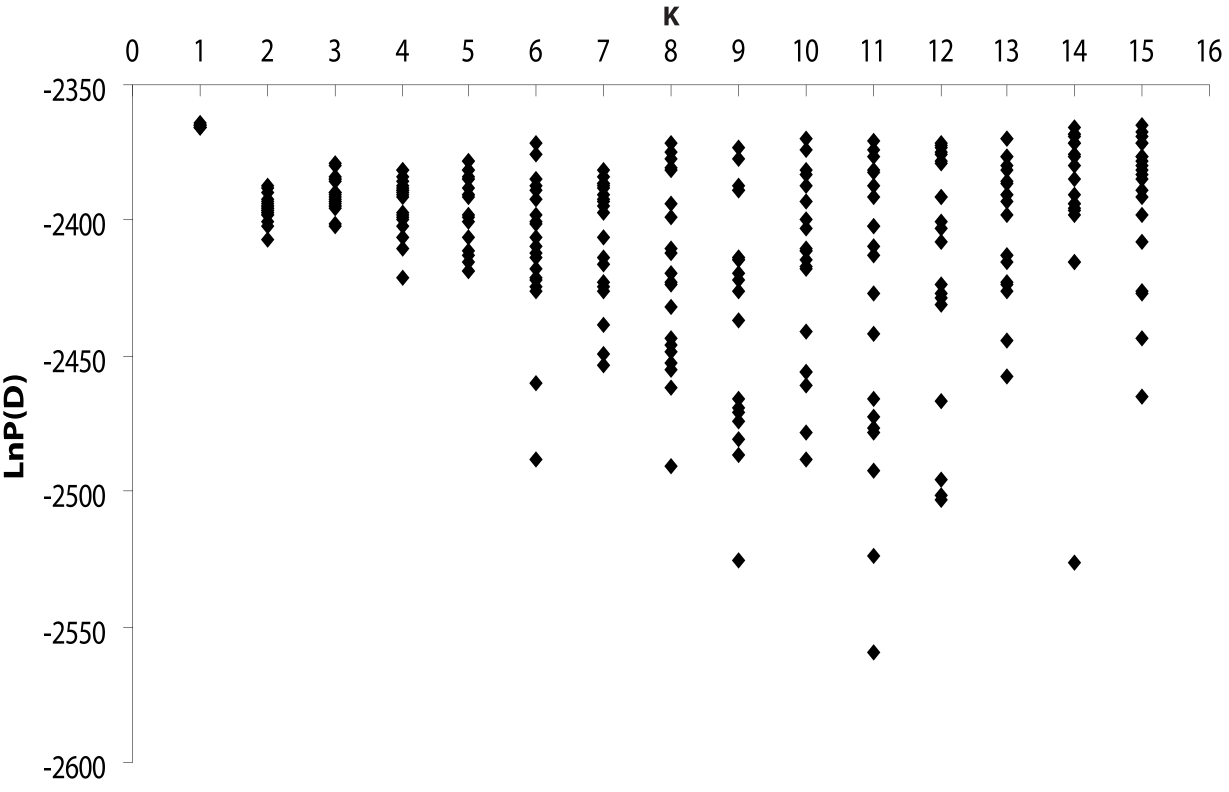

Figure S1. Plot of LnP(D) as a function of the number of clusters (K) across the 20 runs
